# Interpreting cost-effectiveness ratios in a cost-effectiveness analysis of risk-tailored prostate screening: A critique of Callender *et al*.

**DOI:** 10.12688/hrbopenres.13043.2

**Published:** 2020-10-20

**Authors:** James F. O'Mahony

**Affiliations:** 1 Centre for Health Policy and Management, University of Dublin, Dublin, D02 PN40, Ireland

**Keywords:** Cost-effectiveness analysis, cancer screening, incremental-cost effectiveness ratio, prostate screening, PSA

## Abstract

Callender *et al.* recently published a model-based cost-effectiveness analysis of a risk-tailored approach to prostate cancer screening. It considers the costs and effects of prostate cancer screening offered to all men aged 55-69 without any risk selection and, alternatively, over a range of risk-tailored strategies in which screen eligibility is determined by a varying threshold of disease risk. The analysis finds that the strategy of screening men once they reach a 10-year absolute risk of disease of 5% or more is cost-effective in a UK context. I believe there are several problems with the study, mostly stemming from an incorrect interpretation of the cost-effectiveness estimates. I show that one reinterpretation of their results indicates that screening is much less cost-effective than the original analysis suggests, indicating that screening should be restricted to a much smaller group of higher risk men. More broadly, I explain the challenges of attempting to meaningfully reinterpret the originally published results due to the simulation of non-mutually exclusive intervention strategies. Finally, I consider the relevance of considering sufficient alternative screening intensities. This critique highlights the need for appropriate interpretation of cost-effectiveness results for policymakers, especially as risk stratification within screening becomes increasingly feasible.

## Introduction

Callender *et al.* ^1^ recently published a cost-effectiveness analysis (CEA) of a risk-tailored approach to prostate cancer screening. I believe the study’s results are not interpreted appropriately and cannot be considered a reliable guide to prostate screening policy. This commentary explains the problems with the results, examines if they can be usefully reinterpreted, and more generally, attempts to elucidate the issues regarding risk-group selection and the interpretation of cost-effectiveness estimates. The purpose of this commentary is, through critical examination of the Callender *et al.*, to offer guidance to research groups conducting such modelling on how their analyses can best answer policy questions. Secondly, it aims to help readers of such research interpret published estimates.

Callender *et al.*’s ^1^ analysis estimates the total net costs and quality-adjusted life-years (QALYs) of alternative screening approaches. They examine prostate-specific antigen (PSA) based testing every four years between ages 55–69. They consider this strategy when applied to all men within that age range (described as age-based screening) and alternatively, the same strategy starting only when men meet a range of alternative prostate cancer risk thresholds (described as precision screening). They consider 17 alternative risk thresholds ranging from 2% to 10% 10-year absolute risk (10y-AR) in 0.5% increments. Men can reach these thresholds at different ages. This means the men with the greatest total lifetime risk reach any given threshold at an earlier age and the proportion of men having reached any given threshold increases with age. Therefore, relaxing the risk eligibility threshold simultaneously expands the pool of screened men and lowers the age of screening initiation in those screened.

The study reports incremental cost-effective ratios (ICERs) calculated by comparing the total costs and health effects of both age-based screening and the range of precision screening strategies to no screening. The reported ICERs range from £14,862/QALY for the most conservative risk-based strategy that restricts screening to those with a 10y-AR of at least 10% to £34,952/QALY for age-based screening. They report a 10y-AR of 5% yields an ICER of £19,598/QALY and note this would be a cost-effective policy in a UK context in which a cost-effectiveness threshold of £20,000/QALY applies. Their results are presented with caveats regarding the structure of the analytical model used and parameter uncertainty. Callender *et al.*’s ^1^ analysis is a very welcome attempt at examining how prostate screening could be better targeted towards those at greater risk, thereby avoiding unnecessary harms to men at lower risk and enhancing programme cost-effectiveness.

## Critique

This critique addresses three issues. The first relates to differences between the average and incremental effects of lowering the risk threshold of screening. The second concerns the failure to consider mutually exclusive intervention strategies and the implications this has for finding optimal policies for specific risk-subgroups. The third relates to the relevance of varying the intensity of screening for the estimation of ICERs.

### Incremental analysis of risk threshold adjustment

Callender *et al.*’s ^1^ cumulative assessment of the ratio of total costs to total QALYs as the risk threshold is relaxed means the analysis initially includes those men at highest risk who are likely the most cost-effective to screen and then progressively adds those of lower risk that are probably less cost-effective to screen. This cumulative approach to assessing the ratio of total costs to health effects hides the marginal effect of progressively relaxing the risk threshold to include lower risk screenees. The appropriate approach is to examine the incremental change in costs and health effects as the risk threshold is relaxed. Such an incremental analysis identifies what additional health gain is achieved at what additional cost of relaxing the risk threshold relative to the previous, more restrictive threshold.

**Figure 1.**
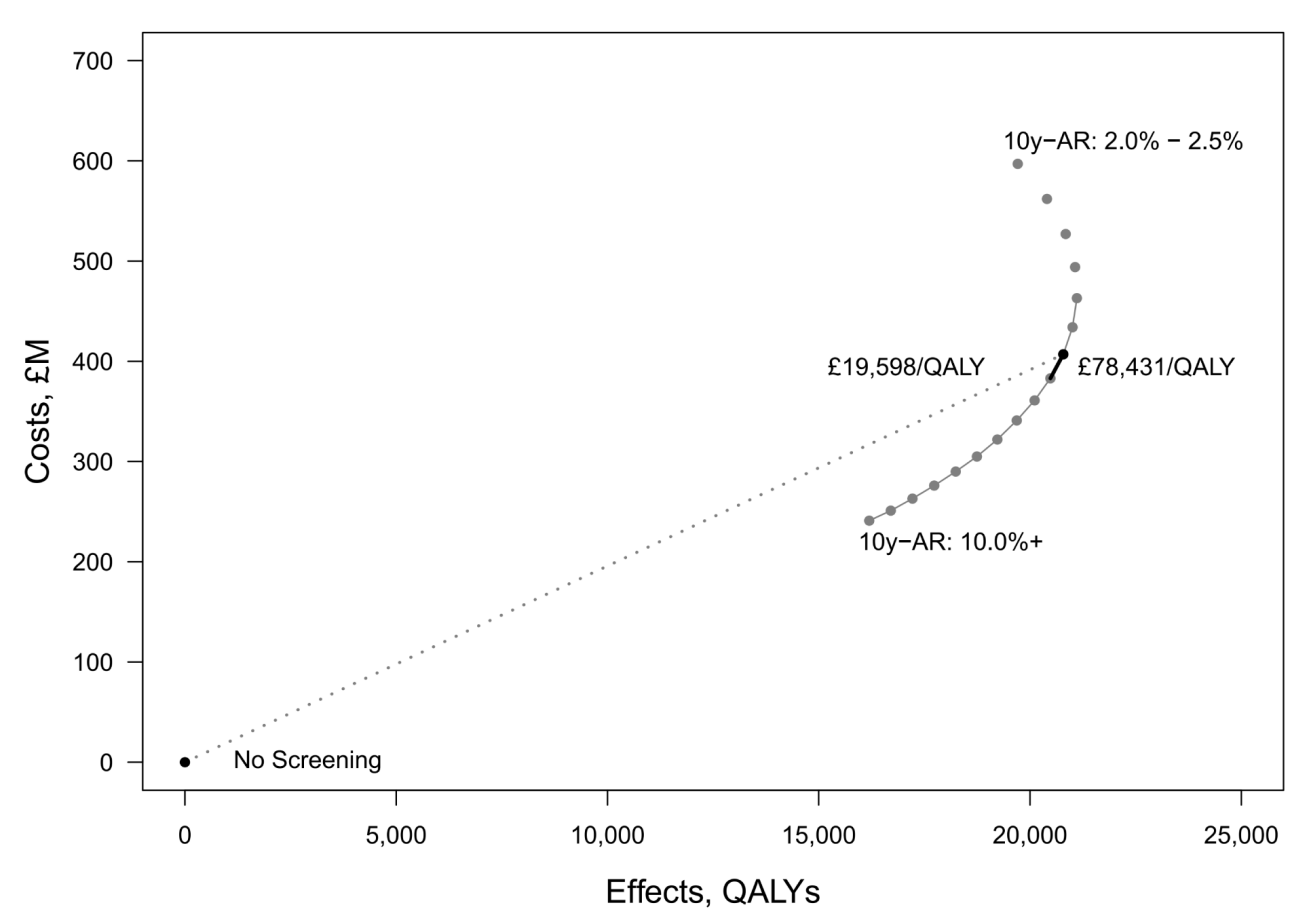
Cost-effectiveness plane demonstrating the difference between cost-effectiveness ratios calculated on a cumulative and incremental basis.

Reinterpreting the results using an incremental approach indicates that relaxing the risk threshold is less cost-effective than appears under Callender *et al.*’s ^1^ cumulative analysis. The difference between the cumulative and incremental appraisal is shown in Figure 1. It plots the estimated costs and effects of the 17 precision screening strategies from Callender *et al.*’s ^1^ analysis. The least costly strategy is for the most conservative risk threshold of 10y-AR of 10%, while the most costly is for the least restrictive 2% 10y-AR threshold. The solid grey line shows the ratio of incremental costs and effects between the risk subgroups. The incremental ratios rise as the risk threshold falls and more men are screened. Beyond a certain threshold total effectiveness falls, implying that some screening becomes harmful to health. That is, it appears that reducing the risk threshold beyond a certain point harms at least some men.

The dotted line in Figure 1 corresponds to the cumulative ratio reported in the by Callender *et al.* ^1^ as an ICER. In this case, corresponding to screening all men with a 10y-AR of at least 5%, which Callender *et al.* ^1^ report to be £19,598/QALY. The ratio of the incremental difference of screening men with a 10y-AR of 5.0–5.5% compared to men with a 10y-AR of 5.5% and above is £78,431/QALY. This ratio is shown in Figure 1 as the single thick black segment of the line joining the strategies. Further detail on the estimate is given in Table 1. It includes the ratios reported Callender *et al.* ^1^ as ICERs (“Reported ICERs”) and an additional cost-effectiveness ratio (CER) calculated as the incremental difference in costs and effects as the risk threshold is incrementally relaxed from 10y-AR of 10% to 2% (“Calculated CERs”). These CERs rise from £14,881/QALY for the highest risk men to £281,553/QALY for men with 10y-AR of 4.0–4.5%. There is no meaningful CER to report below a 10y-AR of 4% once the incremental change in QALY estimates becomes negative.

**Table 1.** Costs, effects and reported ICERs and reinterpreted cost-effectiveness ratios from Callender *et al.*

Strategy: 10yr-AR risk threshold, %	Effects, QALYs	Costs, £M	Reported ICERs, £/QALY	Calculated CERs, £/QALY
No Screening	0	0	-	-
***10.0 +***	***16,195***	***241***	***14,862***	***14,881***
***9.5 - 10.0***	***16,704***	***251***	***15,050***	***19,646***
*9.0 - 9.5*	*17,218*	*263*	*15,281*	*23,346*
*8.5 - 9.0*	*17,732*	*276*	*15,560*	*25,292*
*8.0 - 8.5*	*18,242*	*290*	*15,894*	*27,451*
*7.5 - 8.0*	*18,743*	*305*	*16,289*	*29,940*
*7.0 - 7.5*	*19,227*	*322*	*16,755*	*35,124*
*6.5 - 7.0*	*19,686*	*341*	*17,303*	*41,394*
*6.0 - 6.5*	*20,109*	*361*	*17,947*	*47,281*
*5.5 - 6.0*	*20,482*	*383*	*18,704*	*58,981*
*5.0 - 5.5*	*20,788*	*407*	*19,598*	*78,431*
4.5 - 5.0	21,006	434	20,659	123,853
4.0 - 4.5	21,109	463	21,924	281,553
3.5 - 4.0	21,067	494	23,446	SD
3.0 - 3.5	20,844	527	25,290	SD
2.5 - 3.0	20,401	562	27,542	SD
2.0 - 2.5	19,709	597	30,297	SD
Age-based screening	16,416	574	34,952	SD

Source: Callender *et al*. Table 2. SD: Subject to simple dominance.

The policy significance of the difference between the cumulative and incremental analysis can be seen in the context of the UK cost-effectiveness threshold of £20,000/QALY as referenced by Callender *et al*. ^1^. Using the originally reported cumulative ratios, the first 11 risk thresholds would be considered cost-effective, as their reported ICERs are within the threshold (shown in italics in Table 1). Using the appropriate incrementally calculated CERs however, only the two most restrictive risk categories fall within the threshold (shown in bold). Accordingly, far fewer men appear cost-effective to screen than originally reported. Furthermore, those screened would start at an older age.

As an aside, it is useful to see how my interpretation of the results presented here is supported by the net monetary benefit (NMB) analysis provided by Callender *et al.* ^1^ within a supplementary appendix to their study. The variation of NMB with the risk threshold is presented by them in Figure H (A). It shows that NMB is maximised only when the risk threshold is near its most restrictive around 9.5% to 10% 10y-AR. This contradicts Callender *et al.*’s ^1^ finding that a 10y-AR of 5% would be cost-effective, as NMB should be maximised at the optimally cost-effective strategy. The observed maximisation of NMB around 9.5% to 10% 10y-AR corresponds with the optimally cost-effective policy (within those simulated) when using the incremental interpretation presented here.

### Non-mutually exclusive strategy comparisons

At this point, I now turn to consider can the reinterpreted CERs reported in Table 1 be used as a reliable guide to screening policy. The above critique draws on the long and widely recognised distinction between the average and incremental cost-effectiveness ratios that is reflected in CEA guidelines ^2– 6^. Note, however, that I have not described the CERs in Table 1 as ICERs. This is because there are further complications with the incremental reinterpretation of CERs that means they still may not be considered true ICERs and so are not a suitable guide to policy. Moreover, the published results cannot be reinterpreted into ICERs.

The standard interpretation of an ICER is the incremental comparison of costs and effects of mutually exclusive strategies that lie on the efficient frontier of the cost-effectiveness plane ^7^. The way the alternative policy choices are specified within Callender *et al.*’s ^1^ analysis means they fail to constitute mutually exclusive strategies. As mentioned above, the relaxation of the risk threshold simultaneously adds men of lower lifetime risk of disease to the pool of screened men and reduces the age at first screen for those already within the pool of screened men. Lowering the age of screening initiation in higher risk men is not mutually exclusive of extending screening to lower risk men.

The non-mutually exclusive strategies within Callender *et al.* ^1^ means the incremental CERs reported in Table 1 correspond to a mixture of different policy choices for men of different lifetime risk. For example, reducing the risk threshold will bring forward the age of first screening for men of high lifetime risk, while it may entail a shift from no screening to one or more screens for a man of lower lifetime risk. While both men may have an equal 10y-AR at the initiation of screening, the differences in both their lifetime risk and the number of lifetime screens they receive mean the policy choices will likely be of different cost-effectiveness. There is no way to disaggregate the published results into a form that would permit policy makers to understand how large any differences in cost-effectiveness may be between men of different lifetime risk or identify what the optimal policies would be.

The implication of non-mutually exclusive strategies for policy is that although most of the incremental CERs in Table 1 are above the cost effectiveness threshold, it is not necessarily the case that all the corresponding policies are cost-ineffective. For instance, advancing the age at first screening for a man with a high lifetime risk might be less cost-effective than providing one lifetime screen at age 69 to a lower risk man. Accordingly, it would be premature to base policy on the incrementally interpreted CERs I present in Table 1.

To generate mutually exclusive policies within a single analysis, the authors should have held screening intensity constant for all but one sub-group at a time while varying intensity for the remaining sub-group. This policy generation process would have to be repeated for all sub-groups over all alternative strategies considered, resulting in a very large number of mutually-exclusive strategies. A much simpler alternative would be to model the range of screening strategies in separate analyses for each sub-group according to their lifetime risk.

If Callender *et al.*’s ^1^ analysis were to disaggregate men of different lifetime risks as described, then it would be possible to assess the different simulated screening strategies in each risk subgroup. That would allow the analysis untangle the differences of intensifying screening in higher risk men from extending screening to lower risk men. Such disaggregation is particularly important when we consider that the reduction in the risk threshold is eventually estimated to harm health. It is important to know which men are harmed by what intensity of screening.

### Screening intensity and ICERs

The third issue of ratio interpretation considered here relates to the range of alternative comparator strategies required to adequately estimate ICERs. ICERs give the ratio of the incremental difference in costs to effects between one strategy relative to the next most effective relevant comparator strategy ^7^. For example, the appropriate ICER estimation for a given strategy would typically require comparison to a less intense screening strategy with a lower number of lifetime screens, achieved by comparison to a strategy with either a longer interval or a narrower screening age range ^3, 7^.

Previous prostate cancer screening CEAs demonstrate the relevance of incremental comparisons between alternative screening frequencies and varied screening age ranges. Heijnsdijk *et al.* show how ICERs rise as the number of lifetime screens increases ^8^. While that analysis did not differentiate between risk strata, it does illustrate the relevance of including low intensity strategies as comparators to other strategies with shorter intervals and wider age ranges. They found that in an average risk population in a Dutch context the optimal strategy would be three screens per lifetime at ages 55, 57 and 59. They found no strategy with screening beyond age 59 to be cost-effective, indicating the relevance of considering alternative stopping ages in the case of prostate screening.

This context of previous research and well-established methods guidance tells us that even if Callender *et al.*’s ^1^ results can be disaggregated into mutually exclusive strategies for separate sub-groups according to lifetime risk, the resulting ratios would still only represent incremental changes to the start age of screening. Ideally, we would like to estimate a range of screening intensities in each sub-group, varying not only the screening start age, but also screening interval and, importantly, the screening stop age. In particular, we would be interested in the potential for low intensity screening to offer at least some prevention to the lowest risk men.

Modelling a wide variety of strategies intervals typically requires simulation of the natural history of disease and the imposition of stage-specific estimates test performance characteristics. Not all models are such "deep" models ^9^. Callender *et al.*’s ^1^ analysis apparently is not one such model and may be restricted to the simulation of quadriennial screening intervals used in the trial that informed the model. While analyses limited to the simulation of one screening interval alone may come with the limitations of not being able to estimate a complete ICER on the basis of a comprehensive set of comparator strategies, these limitations are traded off against the advantage of less reliance on assumptions and reduced parameter uncertainty. There is no clear answer on the optimal balance in this trade-off when informing policy. Nevertheless, results from analyses without a complete set of comparators can still be useful to policy makers. CERs from analyses with a limited set of strategies that exceed the cost-effectiveness threshold can usefully rule out strategies as cost-ineffective. This is because any strategy with a CER exceeding the threshold within a limited set of comparators can never be cost-effective within a more complete analysis.

It is important to note that Callender *et al.* did clearly acknowledge the relevance of strategies of alternative age ranges and screening frequencies, but explained the data to support a risk stratified analysis was lacking. Accordingly, while the policy choices they simulated may not be those optimal relative to our theoretical understanding of optimally tailored strategies, the latter are, as yet, unsupported by data. Moreover, we should point towards the usefulness of Callender *et al.*’s analysis in informing further research. Their study concludes by stating prospective randomised controlled trials are required to better inform optimal policy. Further analysis of their model could usefully indicate what strategies would be most useful to compare and which parameters estimates are the priority to refine.

## Discussion

The above critique shows that the ratios reported as ICERs by Callender *et al.* ^1^ should not be used to inform prostate screening policy. Unfortunately there is no way to readily reinterpret the published estimates into policy-relevant guidance. A disaggregation of the model results by the authors could make their analysis more useful to policy makers, but probably only to rule certain strategies out. Further work would be needed to determine if other screening intensities could provide cost-effective screening for lower risk men and what might be the benefit of varying the screening stop age.

Some of the points described in this critique were apparently raised during peer review. The available reviewer comments that accompany the paper show Reviewer 3 noted these issues and explained they could be easily addressed. In reply, the authors give the rationale for their modelling choices, which explain why they did not make the suggested changes. It seems unfortunate that the reviewer’s advice was not considered further as it is evident that the authors have done the hard work of constructing, parameterising and implementing their model. It seems a shame that basic changes were not made prior to publication. This highlights the need for both authors and journal editors to ensure that reviewer comments are adequately accounted for. It also serves as a reminder of the fallibility of the peer-review process.

CEA methods for the analysis of screening are well-established and the need for appropriate ICER comparisons between screening strategies of different intensities has been recognised clearly for many years ^3, 5^. The issues around risk subgroup analysis and how to handle them in screening CEAs have received less attention in the literature ^10– 12^. Given the increasingly nuanced knowledge of risk subgroups provided by research on genetic and other risk factors, it seems likely that risk-stratified analyses such as Callender *et al.*’s ^1^ will become more common. Accordingly, there may be a need for clearer guidelines for analysts.

This commentary is unavoidably critical of the analysis presented by Callender *et al.* ^1^. The intention, of course, is not to single out a single study for criticism. Rather, it is to offer constructive guidance to such modellers on how their analyses can be best specified and interrogated. The question of appropriate strategy comparison is not trivial, especially when variation in disease risk is considered, and Callender *et al.* is certainly not the only study to face pitfalls in this respect. Without clear examination of the problems and clear direction of how they should be avoided, subsequent studies will be prone to error. The research question addressed by the authors is important and deserves attention from health economic modellers. It is hoped that the points raised here may inform a revision of the model and the generation of new cost-effectiveness estimates. Such an analysis would usefully inform the tailored provision of prostate cancer prevention according to disease risk, potentially improving the health of men across the UK and beyond.

The criticism made here of Callender *et al.* reflects a broader tension between pragmatic modelling within the constraints of currently available data among a narrow range of policy alternatives as opposed to the theoretical ideal of modelling many alternative strategies, each considered in disaggregated analyses for separate subgroups. The optimal balance between pragmatism and technical exactitude will always be a matter of debate. We can at least inform this debate by being explicit about the modelling choices made and rationale for them.

## Conclusion

In conclusion, Callender *et al.*’s ^1^ interpretation of their cost-effectiveness estimates is at odds with accepted CEA practice for several reasons. While a reappraisal of their results suggest that quadrennial screening will likely be cost-ineffective for more men than they suggest, it is not advisable to base any policy recommendations on either the originally published results or the illustrative reinterpretation given here. This example is useful in illustrating some of the methodological considerations surrounding the appropriate handling of risk-subgroup specific cost-effectiveness estimates. Such issues of sub-group specific interpretation of evidence are likely to become more prevalent as increasing knowledge of disease processes permits further disaggregation of screen eligible populations.

## Data availability

All data underlying the results are available as part of the article and no additional source data are required.
