# Supplementary material for: Interpreting cost-effectiveness ratios in a cost-effectiveness analysis of risk-tailored prostate screening: A critique of Callender et al
Source: HRB Open Res. 2020 Oct 20;3:23. Originally published 2020 May 13. [Version 2] doi: 10.12688/hrbopenres.13043.2 (PMC7312227; doi:10.12688/hrbopenres.13043.2)
Supplement: Peer review history

# Peer review: Reviewer response for version 2

**DOI:** 10.21956/hrbopenres.14261.r28185

**Published:** 2020-10-21

**Authors:** Eveline Heijnsdijk

**Affiliations:** 1 Department of Public Health, Erasmus MC, University Medical Center Rotterdam, Rotterdam, The Netherlands

I have no further comments.

Are arguments sufficiently supported by evidence from the published literature or by new data and results?

Yes

Is the conclusion balanced and justified on the basis of the presented arguments?

Yes

Is the rationale for commenting on the previous publication clearly described?

Yes

Are any opinions stated well-argued, clear and cogent?

Yes

Reviewer Expertise:

Evaluation of screening.

I confirm that I have read this submission and believe that I have an appropriate level of expertise to confirm that it is of an acceptable scientific standard.

# Peer review: Reviewer response for version 1

**DOI:** 10.21956/hrbopenres.14139.r27411

**Published:** 2020-06-23

**Authors:** Robert Boer

**Affiliations:** 1 Allergan plc., Irvine, CA, USA

The paper by O’Mahony ^1^ critiques another paper by Callender *et al*. ^2^ concerning the cost-effectiveness of prostate cancer screening by estimated risk.

 I agree with each of the three main three lessons of this critique:

1. Consider incremental cost-effectiveness.
2. Consider mutually exclusive intervention strategies.
3. Include varying intensity of screening.

The author explains the lessons well and was able to show that just following lesson 1) would already make a large difference in the concluded policy recommendation, thus showing that the lessons really matter.

 Next to the three lessons that were included in the paper, the author could also have mentioned some other points of critique:

- The Callender *et al.* paper focusses on QALYs as effectiveness measure. Therefore, it seems better to optimize screening based on expected QALYs gained from screening instead of on an estimate of prostate cancer risk. As proxy for QALYs gained, the risk of prostate cancer death seems better than the risk of prostate cancer.
- In order to show the extent of superiority of risk based screening over age based screening, the Callender *et al.* paper should preferably include an efficiency frontier as described by O’Mahony for both age and risk based screening, but at least a scenario that would lead to optimal age based screening at a similar cost as when applying the preferred risk threshold.
- Callendar *et al.*’s estimates for the relationship between risk of cancer, overdiagnosis, and of death, as affected by screening, are quite simplified and should be expected to be considerably less accurate than better models for prostate cancer screening that have been published elsewhere ^3^, and therefore less than state of the art. Application of methods that are well below the state of the art, should probably already preclude informing policy, even before considering the presented results.

Are arguments sufficiently supported by evidence from the published literature or by new data and results?

Yes

Is the conclusion balanced and justified on the basis of the presented arguments?

Yes

Is the rationale for commenting on the previous publication clearly described?

Yes

Are any opinions stated well-argued, clear and cogent?

Yes

Reviewer Expertise:

Health economics.

I confirm that I have read this submission and believe that I have an appropriate level of expertise to confirm that it is of an acceptable scientific standard.

## References

[1] : Interpreting cost-effectiveness ratios in a cost-effectiveness analysis of risk-tailored prostate screening: A critique of Callender et al. *HRB Open Research*.2020;3: 10.12688/hrbopenres.13043.1 10.12688/hrbopenres.13043.1 32596631PMC7312227

[2] : Polygenic risk-tailored screening for prostate cancer: A benefit-harm and cost-effectiveness modelling study. *PLoS Med*.16(12) : 10.1371/journal.pmed.1002998 e1002998 10.1371/journal.pmed.1002998 31860675PMC6924639

[3] : Publications by Cancer Site. *Header - Prostate*.[Accessed - 3rd June 2020; Reference source

# Peer review: Reviewer response for version 1

**DOI:** 10.21956/hrbopenres.14139.r27408

**Published:** 2020-06-09

**Authors:** Eveline Heijnsdijk

**Affiliations:** 1 Department of Public Health, Erasmus MC, University Medical Center Rotterdam, Rotterdam, The Netherlands

This correspondence criticizes a paper of Callender *et al*. at three points: The first point: using average or incremental cost-effectiveness, is a valid point. The optimal strategy, with the ICER just below the cost-effectiveness threshold, will clearly be different. However, in the original analysis, the decreasing QALYs above the 10-year absolute risk threshold of 4% was also stated. The original paper also clearly describes the harms and benefits, which may be more important than the exact value of cost-effectiveness, because the cost-effectiveness threshold is arbitrarily.

 The second and third point (mutually exclusive strategies and range of screening intensities) are also valid points from the cost-effectiveness perspective. This indicates how much additional work is still possible. However, for a first analysis on this subject, the results of the original paper are useful. With all uncertainties in the data, it will be the question if this would be the right moment to perform such an extensive cost-effectiveness analysis.

 Concluding, the points of critique in this correspondence are valid. However, the original paper describes a first exploratory analysis on this subject, which can be extended in future.

Are arguments sufficiently supported by evidence from the published literature or by new data and results?

Yes

Is the conclusion balanced and justified on the basis of the presented arguments?

Yes

Is the rationale for commenting on the previous publication clearly described?

Yes

Are any opinions stated well-argued, clear and cogent?

Yes

Reviewer Expertise:

Evaluation of screening.

I confirm that I have read this submission and believe that I have an appropriate level of expertise to confirm that it is of an acceptable scientific standard.

# Peer review: Reviewer response for version 1

**DOI:** 10.21956/hrbopenres.14139.r27409

**Published:** 2020-06-04

**Authors:** Conor Teljeur, Paul Carty

**Affiliations:** 1 Health Information and Quality Authority, Dublin, Ireland

The paper represents a critique that addresses three key aspects of an original analysis by Callendar *et al*:

1. the differences between calculating average and incremental cost-effectiveness ratios
2. consideration of policymaking implications when modelled comparators do not represent mutually exclusive strategies in a fully incremental analysis
3. the relevance of varying the intensity of screening for the estimation of ICERs.

We will discuss the merits of these critiques individually before providing an overall assessment of the paper.

- ACERs vs ICERs

The deterministic reinterpretation of the results of the original paper, where the strategies are ranked according to total effects and followed by removal of strategies subject to simple dominance (more costly and less effective than the alternative strategy) are then removed, is straightforward. If conducting a fully incremental analysis then this approach should be adopted when interpreting the CERs. The basis for this approach is well-founded in the economic literature, but is often not followed in published economic evaluations. As asserted in the paper, the NMB presented in the original paper supports this re-calculation of the CERs.

- Mutually exclusive strategies

The second characteristic of a fully incremental analysis is that the interventions under evaluation are mutually exclusive (i.e. a patient in one patient group can receive only one of the interventions and this intervention is independent of the intervention received in other patient groups). As described in this paper, the original paper did not model the study populations in separate subgroups. As such, the analysis shifts from a comparison of mutually exclusive options to the consideration of independent programmes, each of which incremental costs and effects have already been calculated against the next best alternative.

 The reasons for options not being mutually exclusive may relate to data availability, computational burden or simply the decision problem posed by the decision-maker. Therefore, this approach can still provide useful information for the decision-maker.

- Varying the intensity of screening

The critique seems to suggest that to adequately estimate cost-effectiveness, the study should consider all reasonable alternative configurations of the prostate screening programme (meaning different start ages, different stop ages, different frequencies, etc). Indeed, in a complete analysis, all relevant comparators will be assessed relative to the next best alternative. However, as per #2 this may not always be feasible due to limitations in relation to data availability. The question posed in the original paper is straightforward: "to assess the balance of benefit and harms, as well as the cost-effectiveness, of the introduction of a polygenic risk-tailored screening programme for prostate cancer." The question is, if you begin screening when someone reaches a certain minimum level of risk (between ages x and y), what is the cost-effectiveness for a given risk cut-off?

 From a policy point of view, the manner in which screening has been assessed in the paper provides useful information: is it prostate cancer screening cost-effective if screening eligibility is decided by risk? It is almost certainly possible to identify variants that are possibly more cost-effective but the complex algorithms associated with them may not be feasible to implement (either because of the difficulties in identifying and calling up individuals, because of the complexity of the public health message, or because of the uncertainty in volumes of people presenting for screening). The paper reduced it down to a simpler question. It is also critical to stress that just because complex pathways for screening can be modelled, it does not mean that the answers are valid or useful. Frequently the parameter data used can only support the analysis conducted on the basis of numerous assumptions with unclear impact on the results. That is, the data for clinical efficacy may be based on a specific frequency or intensity of screening, and when applied to alternative strategies it may have questionable validity. This may be dealt with through uncertainty but that is of limited comfort when the focus is on the “on average” differences.

 It's worth bearing in mind the conclusions stated in the original paper are suitably circumspect: "Based on the results of this modelling study, offering screening to men at higher risk could potentially reduce over-diagnosis and improve the benefit–harm trade-off and the cost-effectiveness of a prostate cancer screening program. The optimal threshold will depend on societal judgements of the appropriate balance of benefits–harms and cost-effectiveness." The phrasing is very cautious and it is clear that the authors are aware of the limitations of their approach. It is not presented as an absolute truth, but merely as pointing the way to the potential for a risk-based approach. It would be helpful if the author could acknowledge this, as otherwise it comes across as an unfairly negative commentary.

 Overall assessment

 Overall, I can’t fault the theoretical logic behind the critique which is well-founded in the economic literature. However, the application in practice is not as straightforward. While the criticisms presented are valid, they are laboured in what would normally warrant only a letter in the journal that published the original article. Decision modellers are faced with the need for pragmatic decision-making according to data availability, technical skillset and decision-maker priorities. When faced with such decisions it is important to consider the relevance of the principle of parsimony (i.e. models are simplifications of reality and should be viewed as such), which is at the core of health economic evaluation. This should be addressed in the paper: modelling does not present the 'truth', it presents a simplification that may or may not approximate the truth. Modelling all possible strategies does not necessarily get you closer to the truth. Indeed, done poorly (even with the best intentions) it may actually get you further from the truth.

 The tone of the critique is quite negative considering that the authors of the original paper appear to have appropriately caveated their findings within their limitations and conclusions. The tone could be amended somewhat by providing an examination of the question in the opening paragraph and acknowledging the efforts of the researchers of the original analysis (as per the latter part of third paragraph).

 As the paper represents a critique of another paper, I think a letter to the journal would be appropriate than a separate publication. The critique would be a lot stronger if it drew on systematic evidence within the economic literature of these issues rather than focusing solely on one paper. This is even more relevant given that the critiques are intertwined to main overall critique: a fully incremental analysis was not performed.

 Finally, the author makes reference to the peer review of the paper, highlighting that one of the issues raised in this paper was also raised in the peer review process of the original paper. It is unfair to draw on specific comments from the peer-review process without a balanced representation of the author’s rebuttal to the comments.

Are arguments sufficiently supported by evidence from the published literature or by new data and results?

Yes

Is the conclusion balanced and justified on the basis of the presented arguments?

Partly

Is the rationale for commenting on the previous publication clearly described?

Yes

Are any opinions stated well-argued, clear and cogent?

Yes

Reviewer Expertise:

Health services research and health economics.

We confirm that we have read this submission and believe that we have an appropriate level of expertise to confirm that it is of an acceptable scientific standard, however we have significant reservations, as outlined above.

# Response

**Published:** 2020-09-04

**Authors:** James O'Mahony

**Affiliations:** Trinity College Dublin, Ireland

**Preface to Replies**

I thank the reviewers for their thoughtful and thorough comments on the previous version of the manuscript. I welcome the opportunity to revise my manuscript and address their points in response.

**Re Comments Regarding Mutually Exclusive Strategies**

Regarding the point that simulation of mutually exclusive strategies may be justified by data constraints, computational burdens or the decision-makers decision problem. We acknowledge here that these may all be valid reasons for specifying an analysis in a certain way. I have included a paragraph at the end of the discussion addressing the tensions between pragmatic modelling with constraints as compared to idealised modelling of all strategies for all subgroups. I have kept this discussion brief and relatively general in order to avoid distracting from the principal focus of the critique.   

**Re Comments Regarding Varying the Intensity of Screening**

The reviewers make a series of very fair points here which I agree with. I have revised my manuscript further to reflect the caveats given by the authors regarding the potential relevance of screening strategies of alternative intensity; to emphasise the relevance of data constraints on simulating multiple strategies; and, to further acknowledge the usefulness of results from a model that might simulate less than the theoretical optimal number of strategies.

**Re "Overall Assessment"**

I fully agree that such a reply would typically be suited to a letter to a journal. Indeed, I wrote to the journal in which the manuscript was initially published (PLOS Medicine) seeking to submit a letter or commentary on the article, but they refused to accept a such submission, instead suggesting that a comment could be added to the article on the journal website. That option seemed inadequate to me as it did not offer the opportunity for a critique to be a fully indexed publication that could be found using research search engines. Furthermore, the proposed option would not offer the opportunity for graphical exposition of the critique. Accordingly, I chose to submit my critique elsewhere.

In part, my response is motivated by my view that a journal publishing work should, as a matter of principle, publish replies to that work. I believe this obligation applies irrespective of whether the journal is an open access journal that charges for publication, as does PLOS Medicine. I consider that obligation important, especially where published work fails to adequately address points raised during peer review, as I believe is the case here.

I understand why the exposition of my critique appears laboured. This is because the simple question of appropriate incremental comparisons is, unfortunately, intertwined with the knotty issue of mutually exclusive subgroups and multiple screening intensities. Had these issues been separate, it would have been possible to offer an incremental reinterpretation of the published results that resolved the problems of a non-incremental analysis. It seemed appropriate that any critique addressing the issue of incremental comparisons should also address the issue of mutually exclusive strategies within the manuscript. To address this latter problem clearly required a more detailed submission.

I fully agree with the observations made by the reviewers regarding pragmatic use of CEA and the limitations that data present. I have not given these concerns full consideration in my initial manuscript. As mentioned above I have now added a paragraph to the discussion to acknowledge these issues regarding the consideration of multiple screening alternatives.

**Re Negative Tone**

I understand that the tone of the critique will have read as negative. I have adjusted both the opening of the manuscript and discussion to better relate the constructive intention of the contribution, which was to explain what was wrong with the published results and to explain how future modelling could to address these issues. In accordance with the reviewers’ recommendation, to further highlight the useful contribution of the authors I have also amended the discussion to note how Callender et al.’s model could usefully inform trial design in order to better guide future policy.

As a footnote on tone, while my submission may be construed as negative, I think it is important that researchers do not shy away from being clear and unequivocal where possible. A direct articulation of what could be changed appears the most positive and constructive way to contribute to the literature as a whole, even if it requires a somewhat uncomfortable examination of a specific study.

**Re Placing Critique in Context of Broader Review of Evidence**

I appreciate that the critique would usefully be placed within a broader review. Indeed, I am currently drafting a manuscript assessing the issue of risk group stratification within screening. However, I felt it was necessary to offer a focused critique of this paper for two reasons. The first is that erroneous findings can endure in the literature if not clearly and promptly challenged on publication. This matters, as such results could be used to inform screening policy, leading to suboptimal allocation of resources. The second is that the issues regarding appropriate strategy specification and comparison are not trivial and wanted to present my critique in sufficient detail for readers to reasonably understand the criticisms made. I do not believe I could offer the level of detail required to fairly assess the published study if it was placed within an analysis of many other studies.

**Re Giving a Fair Reflection of the Authors' Reply to Peer Review**

This is an important point and I would like to address it clearly. I felt the authors’ replies further conveyed a lack of awareness of the relevance of incremental analysis to QALY maximisation and reflected a failure to understand the guidance offered by the reviewer. In reply, they contradict the reviewer’s advice that incremental analysis is the standard approach in economic analysis by asserting comparison to no screening is the current paradigm. When the reviewer states that the policies based on their ACER recommendations would not be cost-effective, the authors assert that they are and would maximise QALYs. Both points are clearly erroneous. Given these replies I do not feel a sense of balance would be enhanced by dwelling on them. Accordingly, I have modified my manuscript simply to note that the authors gave their rationale for not amending their analysis. I feel this is suitably neutral and allows other readers to read the reply to reviewers and come to their own judgement.
